# Positron emission tomography and its role in the assessment of vulnerable plaques in comparison to other imaging modalities

**DOI:** 10.3389/fmed.2023.1293848

**Published:** 2024-02-15

**Authors:** Leonardo Proaño-Bernal, Ana Gilabert-García, Shubhang Sharma-Sharma, Citlali M. Mora-Barrera, Jethro Singer-De-la-Garza, P. Yael Beristain-de-la-Rosa, Martín Roberto Basile-Alvarez, Enrique C. Guerra, Jorge Luis Bermudez-Gonzalez, Santiago Luna-Alcala, Nilda Espinola-Zavaleta, Erick Alexanderson-Rosas

**Affiliations:** ^1^Department of Nuclear Cardiology, National Institute of Cardiology Ignacio Chavez, Mexico City, Mexico; ^2^Faculty of Medicine, National Autonomous University of Mexico, Mexico City, Mexico; ^3^Research Division, Instituto Nacional de Geriatría, Mexico City, Mexico; ^4^Department of Internal Medicine, National Institute of Medical Sciences and Nutrition Salvador Zubirán, Mexico City, Mexico

**Keywords:** PET, USG, MRI, MCTA, vulnerable plaque characterization

## Abstract

The diagnosis and management of vulnerable plaques are topics of high interest in the cardiovascular field. Although imaging techniques like computed tomography angiography (MCTA) and ultrasonography (USG) can structurally evaluate atherosclerotic plaques, they are limited in examining internal cellular processes. Positron emission tomography (PET) molecular imaging, on the other hand, can highlight these cellular processes, including inflammation, angiogenesis, and lipid oxidation. Magnetic resonance imaging (MRI) is also a valuable non-invasive imaging technique that can provide detailed anatomical and functional information on the cardiovascular system. In this review, we compare the advantages and drawbacks of MCTA, USG and MRI imaging techniques with PET molecular imaging in evaluating vulnerable plaques. PET imaging allows physicians to measure different pathophysiological events within the plaque using intravenous radiotracers, of which 18F-fluorodeoxyglucose (18F-FDG) is the most validated one. By using 18F-FDG, physicians can understand the formation of the plaque, assess the accumulation of macrophages, and predict major cardiovascular events. However, some limitations exist in using 18F-FDG, including myocardial uptake and low sensitivity in imaging coronary arteries. We also mention other radiotracers that can help in evaluating vulnerable plaques, including 18F-NaF. Although PET imaging is still challenging, it has shown promise in evaluating vulnerable plaques and could be used to intervene in high-risk patients before major cardiovascular events occur.

## 1 Introduction

Multidetector computed tomography angiography (MCTA) is considered nowadays the imaging technique of choice when addressing non-invasively vascular disease, especially coronary artery disease (CAD) (1). Likewise, ultrasonography (USG) is the imaging modality of choice for evaluating atherosclerotic carotid disease (2). Both first-line imaging techniques have the advantage of being non-invasive, cost-effective, and widely accessible. However, both have several limitations, especially when a comprehensive evaluation of the content and the internal cellular processes occurring in the plaque is relevant for determining the risk of acute vascular obstruction and infarction.

There has been great interest in finding new ways of visualizing not only the anatomical characteristics of atherosclerotic lesions, but also physiological processes that can help ascertain the most likely progression of the lesion. Molecular imaging such as positron emission tomography (PET) could be used to highlight inflammation inside atherosclerotic plaques to predict future acute cardiovascular events, moreover, there are several PET radiotracers that can highlight different cellular processes such as angiogenesis, lipid oxidation, and apoptosis, which most likely may also influence the progression of the lesion (3).

In this review, we aim to compare the most relevant non-invasive diagnostic methods with molecular imaging and highlight the advantages and drawbacks of each of them when evaluating vulnerable plaques (which, due to their structural instability, are prone to generate acute infarctions), as in recent years, PET has risen as a possible and promising contender for vulnerable plaque diagnosis (4–6).

## 2 Pathophysiology of vulnerable plaques

The initiation of atherosclerosis depends on the accumulation of small lipoprotein particles in the intima, particularly at sites of hemodynamic strain, caused by the intake of diets high in cholesterol and saturated fats (1, 7, 8). These particles are susceptible to oxidative stress, which promotes the attraction of leukocytes, particularly phagocytes. After phagocytosis of lipid particles, macrophages become foam cells and adhere to the arterial endothelium by penetrating the endothelial cells. Foam cells embedded within the endothelial layer perpetuate inflammation by promoting the recruitment of more leukocytes via interleukins (1, 8).

Cardiovascular risk factors such as smoking, diabetes, hypertension, and hyperlipidemia induce endothelial dysfunction, reducing the availability of nitric oxide, increasing tissue levels of endothelin 1, and activating pro-inflammatory pathways (8).

Additionally, low and oscillatory shear stress, which is the force exerted by blood flow on the arterial wall, can cause arterial remodeling. This process involves the loss of arterial elasticity, which can result in increased pulse-wave velocity (7). These changes make certain areas of the arteries, such as inner curvatures, branch points, and bifurcations, more susceptible to the development of atherosclerotic plaques. Essentially, these areas are under more stress due to turbulent blood flow that can lead to the initiation and progression of plaque formation (1, 7).

**Figure 1:**
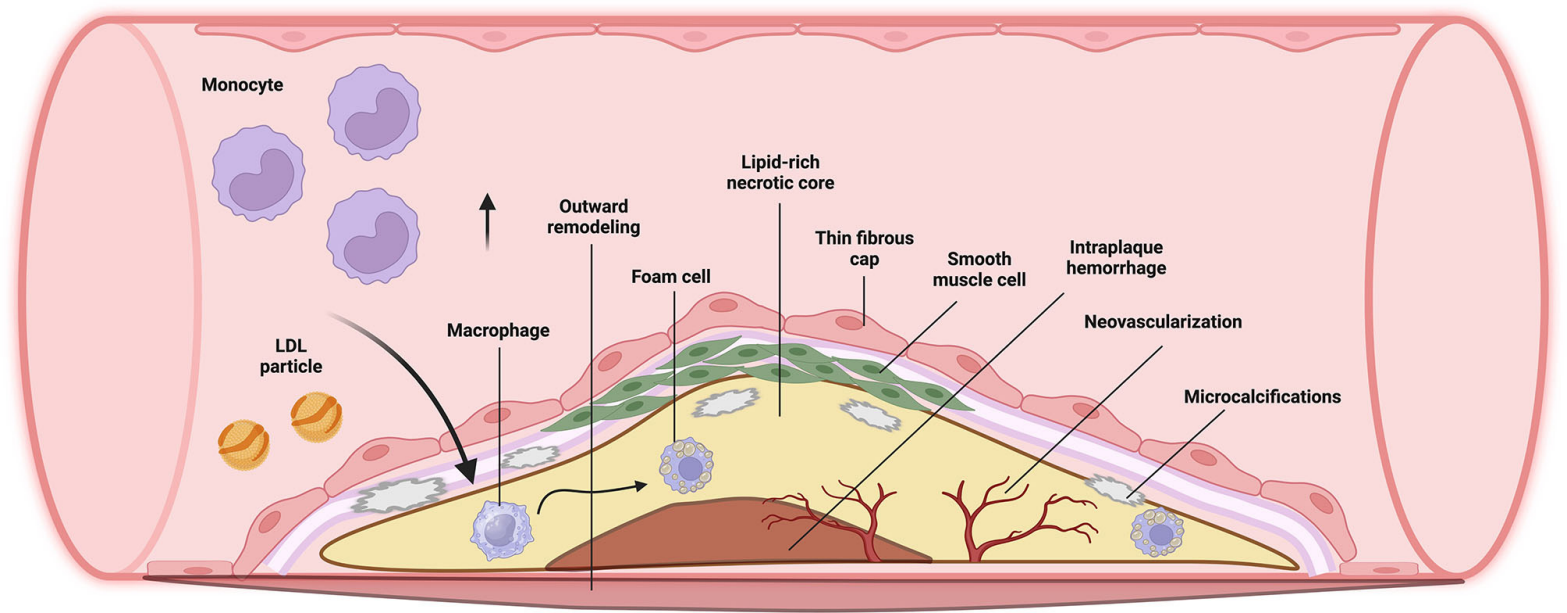
Major components in the formation of vulnerable plaques (1, 8).

Vulnerable plaques have a greater risk of rupturing and triggering the coagulation cascade, leading to cardiovascular events (9). Several studies in patients with myocardial infarction revealed plaque features that make non-obstructive plaques vulnerable to rupture, such as a lipid-rich core (atheroma), a thin fibrous cap, infiltration of inflammatory cells such as macrophages, neovascularization, and spotty calcifications (intra-plaque clusters of calcium), among others (Figure 1) (1, 10).

## 3 Imaging modalities useful for the diagnosis of vulnerable plaques outside of PET

Imaging techniques that evaluate vulnerable plaques can be divided into non-invasive and invasive methods (1). Non-invasive imaging modalities include MCTA, magnetic resonance imaging (MRI), USG, and PET (1). Invasive imaging modalities include intravascular ultrasound, optical coherency tomography and near infrared spectroscopy, these are used after diagnosis is established with non-invasive technics to better characterize plaque elements (1, 11).

Non-invasive imaging modalities are used by physicians to evaluate asymptomatic patients and estimate their risk of cardiovascular events, allowing healthcare professionals to intervene in high-risk patients before major cardiovascular events ensue (12). Many pathological hallmarks of vulnerable plaques can be assessed through these methods, which also have the advantage of establishing risk predictions for major cardiovascular events (5, 13).

### 3.1 MCTA

MCTA is one of the most relevant non-invasive imaging techniques for the assessment of vulnerable plaques, particularly characterized by its good spatial resolution. It can easily estimate lumen stenosis, its morphology, and plaque volume. Moreover, it can detect specific characteristics of vulnerable plaques, such as vessel remodeling (consistent with wall dilation), spotty calcifications, neovascularization, and hemorrhagic, necrotic or lipid rich cores identified by their very low densities (below 30–50 HU) (1, 6, 14, 15).

MCTA is the study of choice for the evaluation of vascular calcifications through the coronary artery calcium score (4). However, the evidence is conflicting, as some authors do not consider calcification as a sign of vulnerability in coronary plaques; and some even suggest it has a role in the stabilization of plaques in older patients (11).

Adding to these, in recent years, new advances within MCTA have been made to ensure that plaque characterization with this diagnostic method is more achievable. Mergen et al. were able to achieve a 0.2 mm resolution, allowing characterization of lipid, fibrotic and calcified components (16).

Due to its lower cost, rapid acquisition time, and more widespread availability, MCTA reaches a wider target population than other invasive and non-invasive techniques (4). However, MCTA has downsides such as difficulty in characterizing fibrous caps, radiation exposure, and a tendency to overestimate the volume of atherosclerosis when compared to invasive imaging techniques (1).

### 3.2 MRI

MRI is another useful imaging technique that does not require ionizing radiation and provides excellent soft-tissue contrast, particularly using non-contrast T1-weighted imaging (black blood imaging) (5, 17). Regarding tissue differentiation, calcification areas can be identified by a loss in signal intensity in every sequence; fibrous tissue by a hypointense signal in all sequences, fibrocellular areas as intermediate to hyperintense areas in all sequences and fatty tissue by a hyperintense signal in T1 with a hypointense signal in T2 (4, 18).

MRI can detect relevant high-risk characteristics in atherosclerotic plaques, such as reduced thickness of fibrous caps or intraplaque hemorrhage. However, it has the disadvantage of having low spatial resolution and being frequently affected by cardiac and respiratory motion artifacts, mainly due to a lower image-acquisition time compared with MCTA and PET, nonetheless, various methods are used in routine clinical practice to correct these artifacts (1).

As a unique feature, MRI can also detect the degree of intraplaque inflammation by using liposome-encapsulated gadolinium (with a hyperintense T1 signal) or superparamagnetic iron oxide (with a hyperintense T2 signal), which are ingested by activated intraplaque macrophages (14, 18). Nonetheless, as mentioned before, it still lacks adequate temporal and spatial resolution when evaluating small vessels or small-sized plaques, like the ones frequently found in coronary arteries, having a negative predictive value of 88.49% when diagnosing CAD (14).

### 3.3 USG

USG is widely used as the first-line imaging method to characterize and assess atherosclerotic disease of the carotids around the world. Numerous ultrasonographic modalities, such as B-mode USG and contrast-enhanced USG, can be used to assess plaque vulnerability (19, 20).

## 4 PET and its role in the evaluation of vulnerable plaques

PET is a hybrid imaging technique used in many clinical situations (5, 21). Despite major advances in nuclear imaging techniques, the evaluation of vulnerable plaques via PET is still challenging (20). PET allows physicians to evaluate different metabolic processes that occur in atherosclerotic plaques through the usage of intravenous radiotracers (13).

Macrophages are the most widely studied element within vulnerable plaques, as they have a very active metabolism and play a fundamental role in the formation and stability of the atherosclerotic plaque (21).

**Figure 2:**
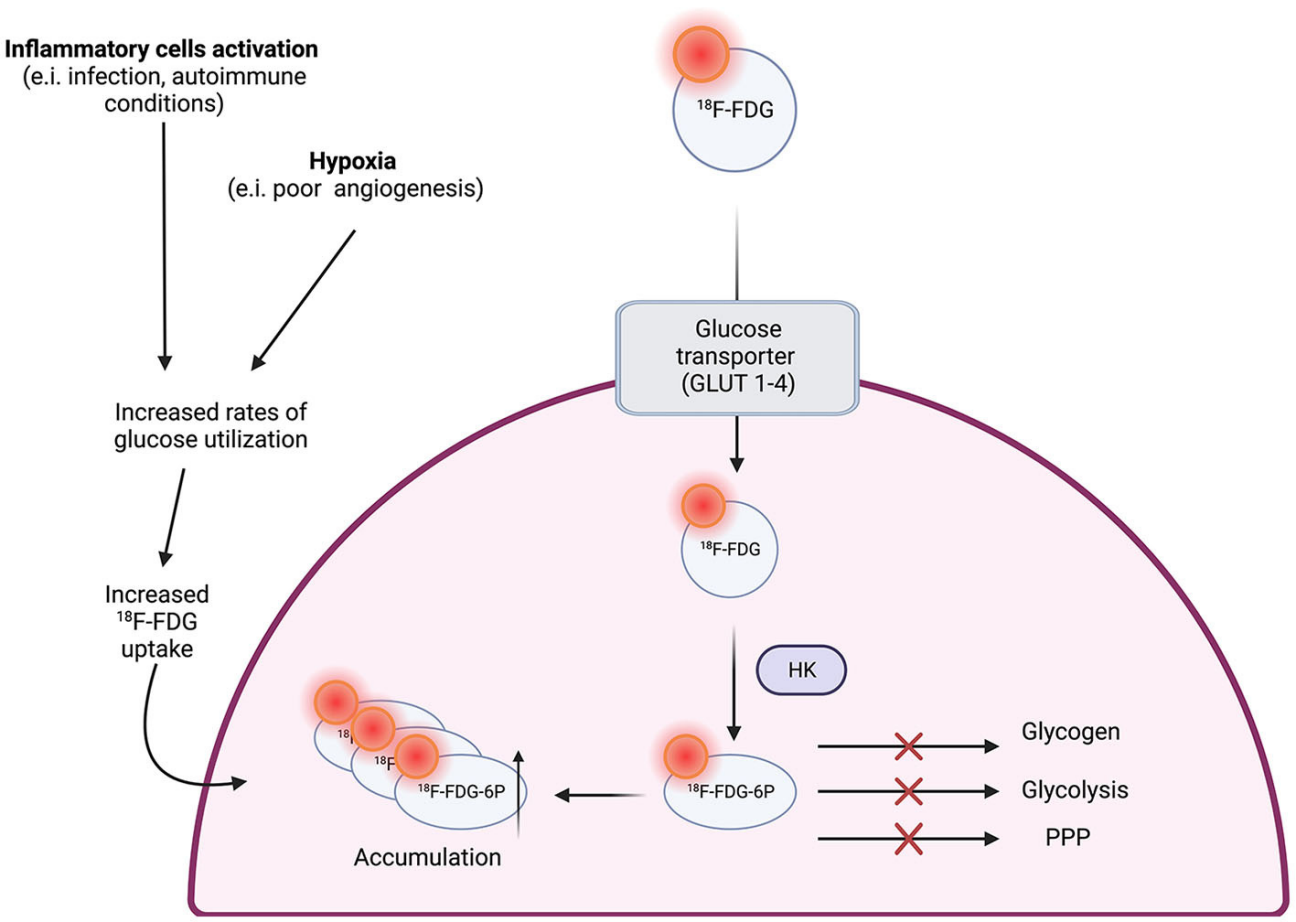
18F-FDG uptake in macrophages (12).

18 F-fluorodeoxyglucose (18F-FDG) is a glucose analog that is captured by metabolically active cells within the atherosclerotic plaque, allowing physicians to achieve a better understanding of plaque formation (Figure 2) (12). It has been used study plaque components and anatomy in the aorta, carotid arteries, coronary arteries, and femoral arteries (5).

Although there is an extensive number of radiotracers available, 18F-FDG is by far the most validated one (12, 21). In multiple studies, it has been associated with the accumulation of macrophages, markers of systemic inflammation, risk factors for atherosclerotic disease, and major cardiovascular events (20). In a study by Figueroa et al., it was proven that arterial activity on 18F-FDG PET images could improve the prediction of future cardiovascular events, including acute coronary syndromes (22).

Despite extensive use of PET, there are still certain limitations when used to evaluate vulnerable plaques, especially when imaging coronary arteries, as glucose uptake is not exclusive to inflammatory cells (5, 12). For example, within the arterial wall, there are other high glycolytic cells that can take up 18F-FDG, dimming the image as non-specific (12). It is also worth noticing that other inflammatory or tumoral processes within the arterial wall, such as arteritis, can affect the sensitivity and specificity of this radio tracer (12, 23).

18 F-sodium fluoride (18F-NaF) is an interesting radiotracer that binds to hydroxyapatite and allows visualization of microcalcifications within the arterial walls; it has been used to identify plaques in the aorta and coronary arteries (5). Because this radiotracer is not taken up by the myocardium, it allows the localization of individual plaques and has demonstrated an excellent inter-observer repeatability (5, 12). It has also been proposed that evaluating the degree of calcification and their location within the atherosclerotic plaque can allow patient stratification based on risk of plaque rupture and disease progression (5, 12, 24).

## 5 PET compared to other non-invasive imaging modalities

### 5.1 PET vs. MCTA

Even if MCTA does not have the capability of detecting relevant metabolic, inflammatory, or angiogenic processes within the plaque, it can provide a more accurate anatomical characterization than molecular techniques. MCTA has a much better spatial resolution (400–600 micrometers) than both MRI (1,300–1,800 micrometers) and PET (3,000–5,000 micrometers) (11). Due to the limitations of spatial resolution of PET, there is a possibility that 18F-FDG uptake in certain diseased artery segments could represent not a vulnerable phenotype of a particular plaque region, but a signal spill-over from adjacent vulnerable plaque segments (~1.5 mm in each direction, proximal, and distal) (25).

In addition to this, the progression of an atherosclerotic plaque from asymptomatic to symptomatic depends on its structure and composition, in which inflammation plays an essential role. PET imaging data of atherosclerotic plaque confirms that the progression of atherosclerosis is a dynamic process with sequential phases of increased inflammation and vascular wall remodeling that can be captured by molecular imaging (26).

A high 18F-FDG uptake in coronary arteries has been linked with the detection of culprit lesions of acute coronary syndrome (*p* = 0.02), which has not been commonly possible to achieve with other frequently used imaging techniques as MCTA (3).

Nonetheless, just like how PET is used to highlight inflammation within atherosclerotic plaques, new computed tomography contrast agents have been created for the same purpose; an example of this is iodinated nanoparticles dispersed with surfactant which has the potential of detecting atherosclerotic lesions rich in macrophages (3). Still, they have not been tested enough and validated for the characterization of vulnerable plaques as PET has.

As mentioned before, 18F-NaF is a common radiotracer used in PET imaging that localizes lesions in the process of microcalcification and inflammation, both of which are commonly seen in the necrotic core of atherosclerotic plaques (27). Although MCTA is known for its usefulness in detecting microcalcification, PET has better sensibility, nonetheless, it suffers from lack of adequate spatial resolution. On the other hand, 18F-NaF doesn't accumulate in more evidently calcified and stable plaques easily detectable by computed tomography (11).

Moreover, high uptake of 18F-NaF in coronary plaques has been associated with accurate determination of culprit lesions when compared to other methods, such as the Agatson score acquired by MCTA (28). Joshi et al. conducted a prospective clinical trial where patients with myocardial infarction and stable angina underwent 18F-NaF and 18F-FDG PET-CT, and invasive coronary angiography in order to determine ratios of culprit and non-culprit coronary plaques of patients with acute myocardial infarction. The authors concluded that 18F-NaF localized recent plaque ruptures in patients with acute myocardial infarction, and in patients with stable coronary artery disease, 18F-NaF uptake seemed to identify coronary plaques with high-risk features on intravascular ultrasound (28).

Furthermore, plaques that are partially calcified, exhibit positive remodeling, have large lipid cores, and spotty calcifications (all features of an unstable plaques) in MCTA, have demonstrated the highest uptake of Na18F in PET imaging. Therefore, the use of 18F-NaF has the capability to identify patients with plaques that may be particularly prone to fissuring or rupturing (23, 28).

### 5.2 PET vs. MRI

Sensitivity and specificity of MRI are very high (20, 29). Unfortunately, MRI pays the price of its high sensitivity and specificity with increased motion artifacts and moderate spatial resolution (15, 20).

According to Morton et al., both PET in combination with computed tomography (sensitivity 82%, specificity 87%) and cardiac MRI (sensitivity 82%, specificity 81%) were effective in detecting CAD (30). Compared to PET, MRI does not expose patients to ionizing radiation and offers outstanding soft tissue contrast (29), but images made with MRI require higher *in vivo* concentrations of labeled molecular probes (15, 29, 31).

The target-to-background ratio is a way of measuring metabolic activity used in PET/18F-FDG to detect macrophage activity (32) in response to hypoxia within an atherosclerotic plaque (23), demonstrating not only inflammation but also probable necrotic areas in the lesion. It has been shown that plaques identified by MRI as lipid-rich in content have a higher mean target-to-background ratio value in PET/18F-FDG than those rich in collagen or calcification, which have a low accumulation of 18F-FDG (*p* < 0.001), demonstrating that lipid-rich plaques are clearly linked to an intra-plaque inflammatory and hypoxic process (33).

There's also a direct correlation between the mean vessel wall area measured by MRI and the mean target-to-background ratio in PET/18F-FDG, demonstrating in this case that there is greater inflammation in plaques associated with a thicker vessel wall (*p* < 0.00001) (33). More recent data have also shown a direct correlation between the number and volume of atherosclerotic plaques, the thickness of fibrous caps, the presence of lipid content, and positive remodeling with the uptake of 18F-FDG in PET (25).

Furthermore, a direct but weak correlation has been exposed between the Ktrans constant measured by MRI (which reflects micro vessel density, microvascular flow, and permeability) and the target to-background-ratio measured by PET/18F-FDG in symptomatic vulnerable carotid plaques (*p* < 0.033). The latter makes sense, as the inflammatory process associated with symptomatic plaques requires higher microvascular permeability for the recruitment of inflammatory cells and the diffusion of inflammatory mediators.

Interestingly, both target-to-background ratio and Ktrans demonstrate an indirect relationship with time since the last ischemic event, showing how the transformation of a plaque into a phenotype of decreased vulnerability reduces the detection of inflammatory mediators (32, 34). Unfortunately, it is not yet clear whether these MRI features (detection of lipid-rich plaques, mean vessel wall area or Ktrans value) can accurately determine inflammation without the use of PET, which is the standard method for assessing inflammatory processes.

While MRI, MCTA, and USG could address hemodynamic factors, PET does not possess this ability (23). Flow-sensitive MRI has shown promising results as a surrogate marker for the detection of vulnerability in atherosclerotic plaques (7).

### 5.3 PET vs. USG

Sang et al. found no overall significant correlations between 18F-FDG PET standardized uptake values (SUV) and duplex-USG echodensity. In this study, no significant correlations were found between lesional grayscale medians (GSMs) and lesional maxSUVs or maxSUV ratios. Notably, there was a correlation between lesional GSMs and lesional maxSUVs in the chronic stenosis group but not in the recently symptomatic stenosis group (35).

Advantages of USG techniques include its low cost, non-invasiveness, availability, intermediate spatial resolution (although not as good as PET, MCTA, and MRI), and radiation-free nature. Unfortunately, some of its limitations are the lack of consistent interobserver and intraobserver agreement, the requirement of image processing techniques, and its poor utility for the characterization of deeply located or small vessels (2, 19, 20).

## 6 Discussion

PET imaging shows promising results as a diagnostic method for plaque characterization. Nonetheless, as reviewed in this article, current non-invasive imaging modalities offer different benefits and approaches to vulnerable plaque evaluation, being the studies of choice when a diagnosis is needed. When choosing between imaging studies for the diagnosis and evaluation of vulnerable plaques, physicians must consider multiple factors, including patient characteristics, the vessel that is being studied, availability, costs, the study's objective, and the need for high resolution images.

PET's advantage lies in its capacity to directly recognize and directly evaluate the pathophysiological process responsible for plaque formation. Let us remember that inflammation within atherosclerotic plaques is fundamental for the evolution of vulnerable plaques (36). The non-invasive imaging techniques are unable to provide the molecular and cellular-level details provided by PET imagining, as they only provide structural characteristics (26).

Since its introduction, multiple studies have demonstrated that PET not only provides information about plaque characteristics but also provides physicians with accurate data to assess prognosis. Rominger et al. demonstrated in their retrospective cohort that patients with previous cardiovascular events and higher radiotracer uptake had a higher risk of developing further events (37).

Despite its obvious benefits, disadvantages such as radiation exposure, the ability to obtain or produce radiotracers, availability, and costs, limits the clinical scenarios in which PET is used for the evaluation of vulnerable plaques. It's also important to remember that in certain patients, such as those being evaluated for coronary disease, uptake of radiotracers by other tissues diminish the sensitivity and specificity of the study (38, 39). New hybrid technologies (such as the conjunction of MRI and PET) promise to tackle spatial resolution disadvantages (40, 41), moreover, as mentioned already in this review, MCTA and PET can be used together to enhance their advantages.

PET's future as a diagnostic method seems to be deeply intertwine with its capacity to recognize plaques prone to rupture. Patients with high-risk lesions within arteries, especially in the coronaries, are the most obvious candidates for the usage of PET, as they could benefit from early detection of vulnerable plaques and receive early invasive interventions. Nonetheless, diagnostic methods such as MCTA, continue to be the gold standard for plaque evaluation. Even though multiple studies mentioned in this review have shown promising results with PET and certain radiotracers, there are multiple imaging resolution artifacts and logistical inconveniences (such as acquisition of radiotracers and high PET costs) that must be addressed before PET becomes a more widely used method for vulnerable plaques diagnosis.

As new radiotracers and advances in spatial resolution emerge, and costs of PET imaging become more accessible, the evaluation of vulnerable plaques via this method will become more widely used by physicians all around the globe. Until then, more studies are needed to standardize the implementation of PET for the diagnosis of vulnerable plaques.

## Author contributions

LP-B: Conceptualization, Investigation, Methodology, Project administration, Supervision, Writing – original draft, Writing – review & editing. AG-G: Investigation, Writing – review and editing. SS-S: Investigation, Writing – review & editing. CM-B: Investigation, Writing – review and editing. JS-D-l-G: Investigation, Writing – review & editing. YB-d-l-R: Investigation, Writing – review & editing. MB-A: Investigation, Writing – review and editing. EG: Investigation, Writing – review & editing. JB-G: Investigation, Writing – review & editing. SL-A: Investigation, Writing – review & editing. NE-Z: Investigation, Writing – review & editing. EA-R: Conceptualization, Investigation, Methodology, Supervision, Writing – original draft, Writing – review & editing, Project administration.
